# A Compact Dual-Port Dual-Polarized Ultrawideband Wearable Textile Antenna for Off-Body Communications in IoT-Based WBAN Scenarios

**DOI:** 10.3390/s26154863

**Published:** 2026-08-02

**Authors:** Kun Guo, Xiang Gao, Wenfei Tang, Xiangyuan Bu, Jianping An

**Affiliations:** 1 School of Cyberspace Science and Technology, Beijing Institute of Technology, Beijing 100081, China; guok@bit.edu.cn (K.G.); an@bit.edu.cn (J.A.); 2 School of Information and Electronics, Beijing Institute of Technology, Beijing 100081, China; tangwf@bit.edu.cn (W.T.); bxy@bit.edu.cn (X.B.)

**Keywords:** compact, dual-port, dual polarization, IoT-based WBAN, off-body communications, ultrawideband, wearable textile antenna

## Abstract

This article proposes, to the best of our knowledge, the first dual-port compact dual-polarized ultrawideband wearable textile antenna covering lower UHF bands for off-body communications in Internet-of-Things-based wireless body area network (IoT-based WBAN) scenarios. The antenna covers key bands for diverse services, including the 470–510 MHz LoRa WAN, 700 MHz offline emergency communication, 900 MHz NB-IoT, and 1–1.2 GHz satellite internet bands. The antenna adopts a square-ring loaded wide slot structure and a multi-mode resonant feeding structure to achieve ultrawideband operation. Moreover, it utilizes oppositely placed advanced microstrip feeding networks to excite the horizontal and vertical polarization modes, respectively, and four narrow slots around the wide slot to extend the current path, thus enabling a compact size of 0.30 × 0.28 × 0.0035 *λ*_l_^3^ (where *λ*_l_ is the largest operating wavelength). Measured −10 dB impedance bandwidths are 119.1% (0.35–1.38 GHz) for Port 1 and 115.9% (0.39–1.37 GHz) for Port 2 on the human body, with more than 19 dB port isolation over the operating band. The measured average gains are about 4.21 dBi for Port 1 and 3.54 dBi for Port 2 on the human body, respectively. Specific absorption rate analysis confirms compliance with the IEEE C95.1 limit at 0.5 W input power. Wireless transmission experiments at IoT bands further validate reliable off-body links with excellent signal-to-noise ratios for both polarizations. The antenna shall be very attractive for off-body communications in IoT-based WBAN scenarios.

## 1. Introduction

As the core of the new generation of information technology, the Internet of Things (IoT) achieves intelligent collaboration among humans, devices, and the environment through ubiquitous interconnection, profoundly reshaping the modes of information exchange and automated operation. Within the diverse application ecosystem of IoT, wireless body area networks (WBAN) stand out as a highly representative and critical scenario [1,2,3]. The rapid development of body-centric wireless communication systems has posed urgent demands for communication stability and body adaptability. In such systems, the antennas serve as the core interface between the transceivers and networks, undertaking the key functions of signal transmission and reception and enabling numerous key applications of IoT-based WBAN. With the advancement of material science and manufacturing technologies, wearable antennas are distinct from traditional rigid and bulky ones, which have been extensively studied and have found applications across diverse fields including health monitoring [4,5,6], emergency rescue services [7,8,9,10], positioning and navigation [11,12], and shared economy scenarios [13,14,15]. The integration of modern wearable antennas has notably enhanced the connectivity, adaptability, and user experience under the framework of IoT-based WBAN.

As demands grow for simultaneous and dynamic access to the IoT by multiple users across various scenarios, antennas capable of supporting multisystem high-rate data transmission and mitigating polarization mismatches have become critical requirements for IoT-based WBAN, especially for off-body communication links [16,17,18] that underpin most cross-scenario interactions. For such off-body links, wideband and ultrawideband operation is essential to cover the multi-protocol frequency bands required by heterogeneous application scenarios and enable high-throughput data transmission. In particular, the low UHF band, such as the 470–510 MHz LoRa WAN and 900 MHz NB-IoT bands, is of paramount importance for IoT-based WBAN applications owing to their superior propagation characteristics, including longer communication range, better penetration through obstacles, and lower path loss, which are highly desirable for outdoor off-body communication scenarios. Therefore, extending wearable antenna operation to these sub-1 GHz bands while maintaining wideband coverage across higher frequency bands is a pressing need for next-generation IoT-based WBAN systems. Meanwhile, polarization diversity can effectively offset polarization mismatches caused by dynamic posture changes of wearable users during movement. In response to these scenario-specific demands, the design of wearable antennas equipped with wideband operation and polarization diversity is very much desired and thus the first critical consideration. Besides, wearable antennas deployed on the human body face additional design challenges. Beyond satisfying fundamental performance requirements, wearable antennas must also address structural deformation triggered by human motion [19,20,21,22]. A compact design not only effectively mitigates human motion-induced structural deformations under dynamic wearable conditions but also enables seamless integration with clothing, thus preventing such distortions while enhancing the overall comfort and flexibility of wearable devices. As a result, leveraging a compact and lightweight design to ensure reliable performance under dynamic wear scenarios has emerged as a critical hurdle in wearable antenna designs. Lastly, to ensure secure operation of wearable antennas in IoT-based WBANs, minimizing the specific absorption rate (SAR) is another key design consideration [23,24,25]. In summary, developing a wearable antenna that integrates wideband operation covering critical sub-1 GHz IoT bands, polarization diversity, compact size, and acceptable SAR levels to meet the simultaneous requirements of supporting multisystem high-rate data transmission and mitigating polarization mismatches has emerged as a major hurdle for off-body communications in IoT-based WBAN scenarios.

In recent literature, most research still faces prominent technical bottlenecks in achieving multisystem high-rate data transmission and polarization diversity, failing to integrate ultrawideband operation, polarization diversity, and compactness. To meet the multisystem access requirements, most existing designs adopt multiband or wideband wearable antennas. Multiband wearable antennas [26,27,28,29,30,31,32,33,34,35,36] are mainly realized through fractal geometries. Wideband and ultrawideband wearable antennas [37,38,39,40,41] are developed via optimized radiating structure designs. However, these approaches have inherent limitations despite their preliminary ability to cover multiple communication bands. Multiband designs suffer from relatively narrow sub-band bandwidths that cannot fully meet the demands of high-rate data transmission. Multiband wearable antennas are also highly susceptible to resonance detuning effects induced by body loading and bending effects during wear, leading to unstable communication performance. For wideband and ultrawideband designs, although substantial bandwidth enhancement is achieved, such antennas remain inherently constrained by single-polarized operation. This makes them vulnerable to polarization mismatches caused by dynamic human body movements. Furthermore, they generally feature large physical sizes and bulky structures that are incompatible with compactness and wearability requirements. Meanwhile, multiple-polarized [42,43,44,45] and circularly polarized [46,47,48] wearable antenna designs have gained attention, which effectively mitigate posture-induced polarization mismatches and improve link stability. However, these designs generally suffer from narrow bandwidths that fail to meet multisystem high-rate transmission demands. Their multi-polarization operation also relies on complex multilayer structures, leading to relatively high profiles and large sizes that compromise wearability and contradict the compactness requirements, especially for lower band applications. It is worth noting that the vast majority of existing wearable antenna designs predominantly operate at frequencies above 1 GHz (e.g., 2.4/5.8 GHz ISM, 5G bands). Research on wearable antennas covering the low UHF band remains remarkably scarce [7,49,50,51,52], despite the critical importance of these bands for long-range low-power IoT communication scenarios. Overall, the absence of a design integrating ultrawideband, multi-polarization, and compactness underscores the imperative of developing a novel wearable antenna for off-body communications in IoT-based WBAN scenarios.

**Figure 1:**
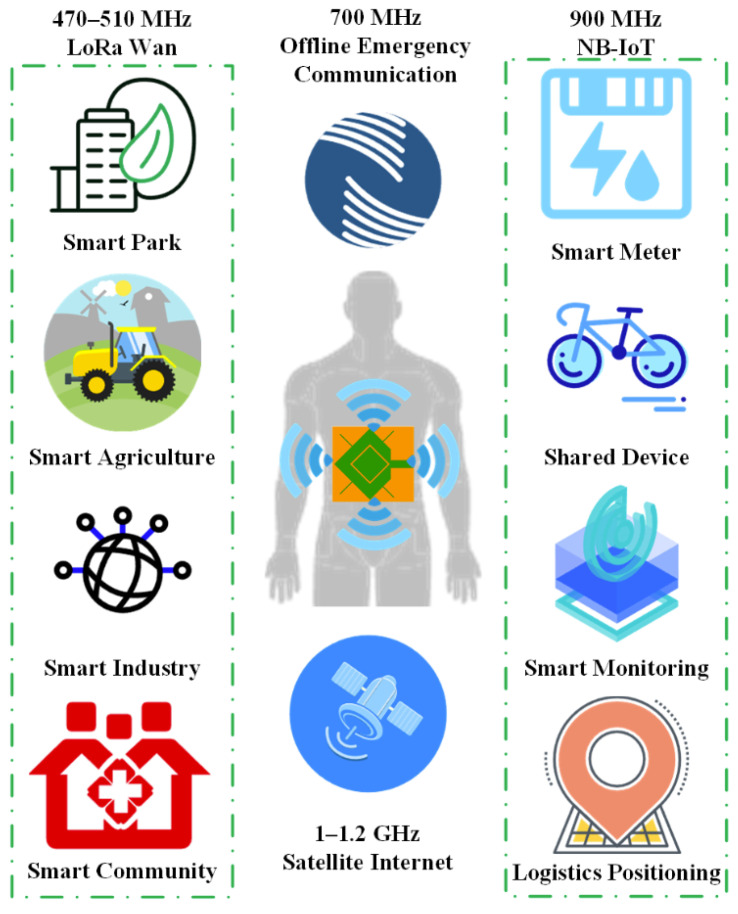
Conceptual illustration of applications for the proposed wearable textile antenna in IoT-based WBAN scenarios.

This article proposes, to the best of our knowledge, the first compact dual-port dual-polarized ultrawideband wearable textile antenna covering lower UHF bands for off-body communications. As shown in Figure 1, the proposed wearable antenna covers key bands for diverse services, including the 470–510 MHz LoRa WAN, 700 MHz offline emergency communication, 900 MHz NB-IoT, and 1–1.2 GHz satellite internet bands in IoT-based WBAN scenarios. The innovation of this work lies in the first-time joint adoption of a square-ring loaded wide slot structure and a multi-mode resonant feeding structure, which excites and merges multiple resonant modes to achieve ultrawideband operation without the bulky geometries of conventional tapered or fractal feeds. Oppositely placed monopole-like and meandered microstrip lines are utilized to realize dual linear polarizations and high isolation, while the etching of orthogonal rectangular slots is used to achieve size miniaturization with an extremely low profile. Additionally, the SAR level is verified to comply with the limits specified in IEEE C95.1 at an input power of 0.5 W. Those performance results have demonstrated that the proposed wearable antenna can be potentially applied in off-body communications in IoT-based WBAN scenarios. The main contribution of this work is that the proposed antenna achieves the following five synergistic breakthroughs.

1. Ultrawideband operation with impedance bandwidths exceeding 115% for both ports in the low UHF band.
2. Dual-linear polarizations, maintaining port isolation above 19 dB.
3. A compact size with a small area of 0.30 × 0.28 *λ*_l_^2^ and a low profile of 0.0035 *λ*_l_ (where *λ*_l_ is the largest operating wavelength), achieving 36% size reduction as compared with the wide-slot radiator.
4. Adaptability to body loading and converting the quasi-omnidirectional beam into a unidirectional beam with a front-to-back ratio (FBR) of more than 17 dB.
5. Wireless transmission quality validated through off-body link experiments at representative IoT bands, confirming excellent signal-to-noise ratios for both polarizations.

## 2. Antenna Design and Analysis

### 2.1. Antenna Configuration

**Figure 2:**
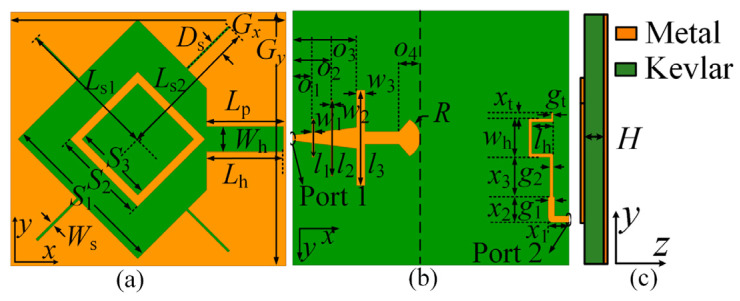
Antenna configuration: (**a**) top layer; (**b**) bottom layer; (**c**) side view.

The configuration of the proposed antenna is shown in Figure 2. All the metallization patterns use the flexible conductive fabric with surface resistance of 0.05 Ω/sq, which are designed on both sides of the Kevlar substrate with thickness of 3 mm. Kevlar fabric is a type of flexible textile dielectric with relative permittivity of *ε_r_* = 2 and loss tangent of tan*δ* = 0.02, which was measured according to the method reported in [53]. The top layer is composed of a ground plane etched with a square-ring loaded modified wide slot, where four narrow slots are orthogonally etched around the wide slot for miniaturization. The bottom layer consists of two advanced feeding microstrips, one of which is a monopole-like structure generating *x*-direction polarization, and the other is a meandered structure exciting *y*-direction polarization. The fan-shaped branch and three parallel-placed rectangular branches of the monopole-like microstrip line are designed to generate new resonances and improve impedance matching. The meandered microstrip line excites the rectangular slot etched on the top layer of the modified wide slot, and this geometry can effectively achieve a broad bandwidth. Ports 1 and 2 are both connected to the external 50 Ω coaxial cables. The optimized parameters of the proposed antenna obtained through parametric analysis are as follows: (parameters’ dimensions in millimeters) *G_x_* = 260, *G_y_* = 240, *S*_1_ = 160, *S*_2_ = 90, *S*_3_ = 75, *L*_p_ = 75, *L*_h_ = 72.5, *W*_h_ = 24, *W*_s_ = 2.5, *L*_s1_ = 135, *L*_s2_ = 150, *D*_s_ = 30, *R* = 20, *o*_1_ = 18, *o*_2_ = 36, *o*_3_ = 60, *o*_4_ = 20, *w*_1_ = 2, *w*_2_ = 2.5, *w*_3_ = 8, *l*_1_ = 36, *l*_2_ = 72, *l*_3_ = 90, *R* = 20, *g*_1_ = 6.4, *g*_2_ = 3, *g*_t_ = 1, *x*_1_ = 15, *x*_2_ = 24, *x*_3_ = 37.5, *x*_t_ = 10, *w*_h_ = 36, *l*_h_ = 20.

### 2.2. Design Evolution Process

**Figure 3:**
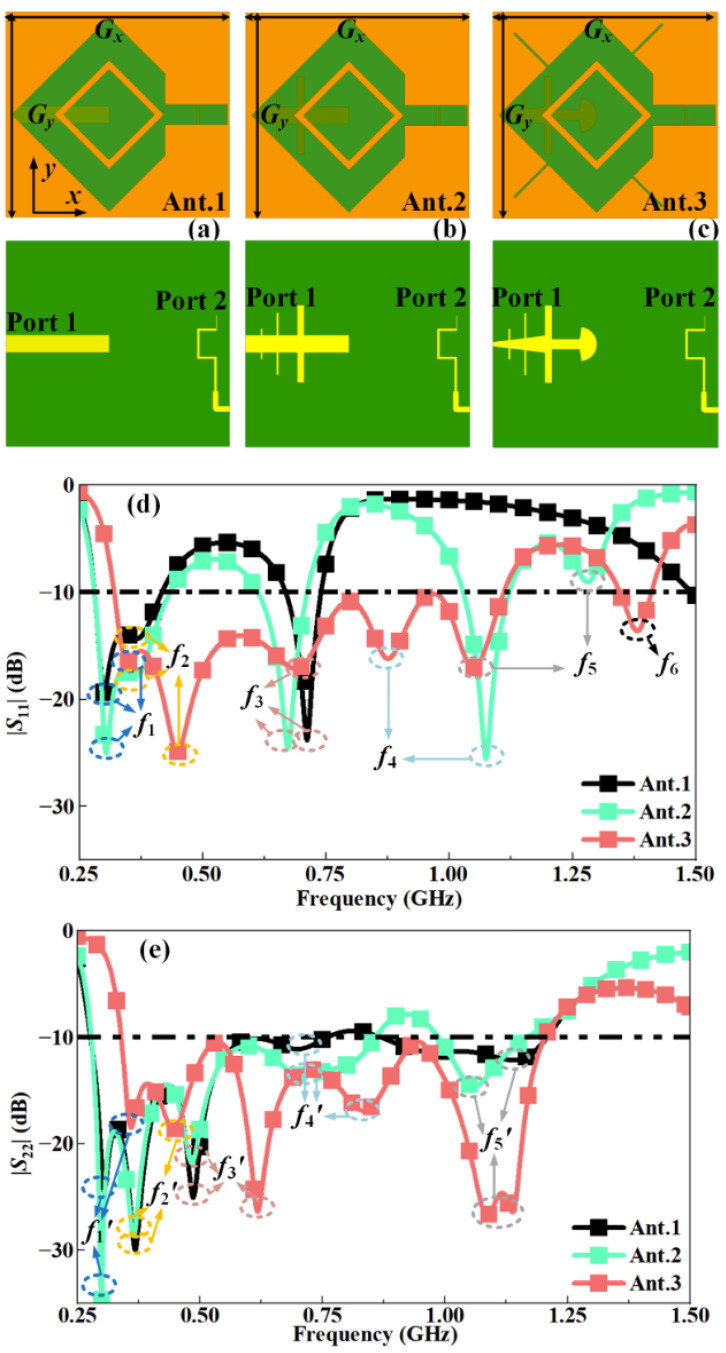
Evolutional designs of the antenna: (**a**) a square-ring loaded wide slot fed by the straight and meandered microstrip lines, with *G_x_* × *G_y_* = 0.375 × 0.35 *λ*_l_^2^; (**b**) a square-ring loaded wide slot fed by a monopole-like microstrip line with parallel branches and meandered microstrip line, with *G_x_* × *G_y_* = 0.375 × 0.35 *λ*_l_^2^; (**c**) a square-ring loaded wide slot with four etched narrow slots fed by advanced microstrip lines, with *G_x_* × *G_y_* = 0.30 × 0.28 *λ*_l_^2^; (**d**) simulated |*S*_11_|; (**e**) simulated |*S*_22_|.

Figure 3a–c show and detail the design evolution of the proposed antenna. Figure 3d,e show the simulated |*S*_11_| and |*S*_22_| of Ant.1, Ant.2, and Ant.3. For clarity, homogeneous resonances for each evolutionary stage are marked with the same color as shown in Figure 3d,e.

As shown in Figure 3a, Ant.1 has a dimension of 0.375 × 0.35 *λ*_l_^2^. Straight and meandered microstrip lines are used as the feeding structures for Ports 1 and 2, to excite the x-direction and y-direction polarizations, respectively. Such opposite placement of those two ports helps to effectively enhance port isolation. The partially deformed wide slot produces a lower resonant frequency corresponding to the fundamental mode at *f*_1_ for the Port 1 excited mode and *f*_1_′ for the Port 2 mode (both around 0.30 GHz). Additionally, another resonant mode at *f*_2_ and *f*_2_′ (both around 0.37 GHz) in the vicinity of the fundamental mode can be excited by the 45-rotated modified wide slot. Furthermore, the square-ring at the center of the wide slot introduces an additional intermediate resonant frequency at *f*_3_ (around 0.70 GHz) for the Port 1 mode and *f*_3_′ (around 0.50 GHz) for the Port 2 mode. Additionally, it is worth noting that the meandered microstrip line excites two higher resonant frequencies at *f*_4_′ (around 0.75 GHz) and *f*_5_′ (around 1.10 GHz) for the Port 2-excited mode, respectively.

Compared to Ant.1, Ant.2 and Ant.3 utilize an advanced monopole-like microstrip line with three parallel rectangular branches in the *y* direction to generate three higher resonant frequencies at *f*_4_ (around 1.10 GHz), *f*_5_ (around 1.30 GHz), and *f*_6_. Due to the short electrical length of the branch with length of *l*_1_ and width of *w*_1_, its contributed resonance *f*_6_ lies outside the operating frequency band but helps with bandwidth optimization. All three *y*-direction branches synergistically improve the impedance matching. It should be mentioned that Ant.3 incorporates four narrow slots etched around the wide slot, which effectively extends the current path and reduces the antenna’s overall dimensions to 64% of the area of Ant.2 (i.e., 0.30 × 0.28 *λ*_l_^2^). Simultaneously, impedance matching for the Port 1 excited mode is further enhanced by adopting a tapered-width monopole-like microstrip line integrated with a fan-shaped branch. Additionally, no new resonances are generated for the Port 2 excited mode throughout the evolutional design process, and thus only impedance optimization is performed for the Port 2 mode.

To further clarify the independent contributions of each structural component to the overall performance, the functional roles of key structures are summarized based on the above evolution analysis. First, the square-ring loaded wide slot serves as the fundamental radiator, which determines the low-order resonant modes and lays the foundation for dual-polarized radiation capability and initial bandwidth. Second, the multi-mode resonant feeding structures (the monopole-like feed with parallel branches for Port 1 and the meandered feed for Port 2) excite orthogonal polarization modes respectively, introduce additional high-frequency resonant modes to extend the upper bandwidth limit, and optimize impedance matching across the entire operating band. Third, the four orthogonally etched narrow slots around the wide slot achieve a 36% area reduction by extending the surface current path, with negligible impact on resonant modes and port isolation. Fourth, the oppositely placed feeding configuration spatially separates the current paths of the two polarization modes, which is the core mechanism for achieving port isolation higher than 19 dB across the full band.

### 2.3. Simulation Analysis

**Figure 4:**
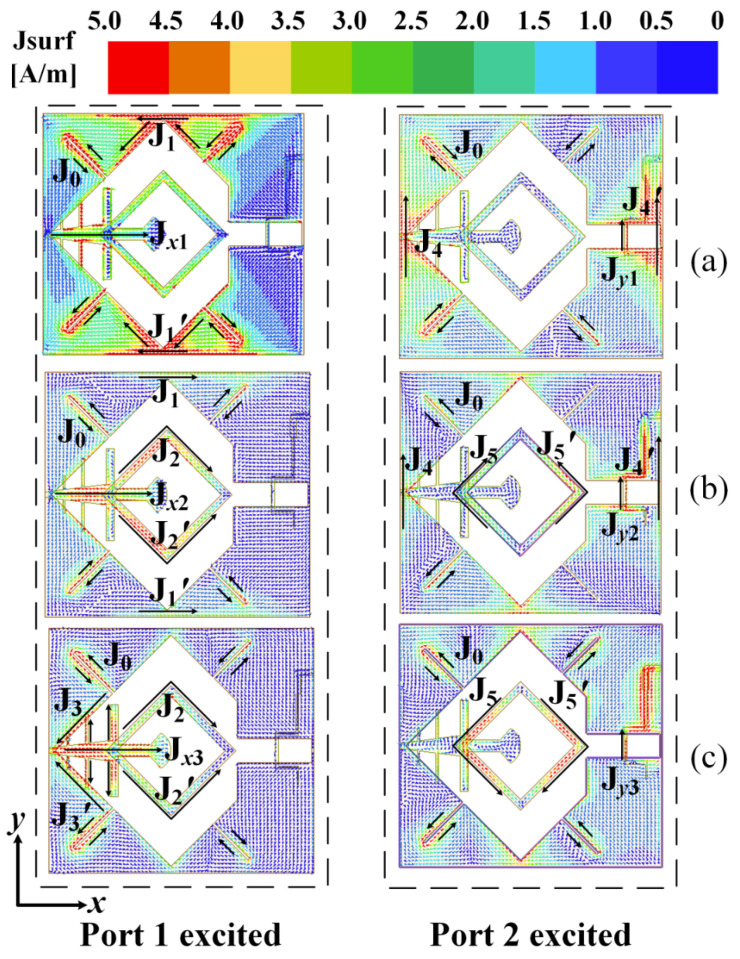
Simulated current distribution of the proposed antenna at different operating frequencies: (**a**) 0.35 GHz; (**b**) 0.65 GHz; (**c**) 1.15 GHz.

To further clarify the operating mechanism of the proposed antenna, simulation analysis is conducted using the software ANSYS High Frequency Structure Simulator (HFSS 2022 R1). Figure 4 shows the current distribution of the antenna at three different operating frequencies. Figure 4a shows that when Ports 1 and 2 are excited separately, the currents **J**_1_, **J**_1_′, **J**_4_, and **J**_4_′ mainly contribute radiation in the *x* direction and *y* direction at 0.35 GHz, respectively. Among them, **J**_1_ and **J**_1_′, which are distributed at the right-angle portions of the wide slot, have the same current components along the *x* direction and opposite components along the *y* direction. Figure 4b shows that additional currents **J**_2_, **J**_2_′, and **J**_5_, **J**_5_′ are generated by the square-ring at the operating frequency of 0.65 GHz. **J**_2_ and **J**_2_′ have the same current components along the *x* direction and the opposite current components along the *y* direction. Similarly, **J**_5_ and **J**_5_′ have the same current components along the *y* direction and the opposite current components along the *x* direction. Therefore, the main polarization modes are still kept. When the operating frequency is further increased to 1.15 GHz, as shown in Figure 4c, the situation for Port 1 excitation becomes such that the generation of **J**_3_ and **J**_3_′ forms a dipolar current mode with **J**_2_ and **J**_2_′, whose current components in the *y* direction can still basically cancel out. For Port 2 excitation, the amplitudes of **J**_5_ and **J**_5_′ are significantly improved, while **J**_4_ and **J**_4_′ are essentially reduced. It should be noted that the currents along the four narrow slots placed orthogonally in the wide slot are in reverse directions and basically have the same magnitude over the operating frequency band. In addition, since the feeding networks are basically separated from the ground, **J***_x_*_1_, **J***_x_*_2_, and **J***_x_*_3_, as well as **J***_y_*_1_, **J***_y_*_2_, and **J***_y_*_3_, also contribute part of the radiation. Note that even though the branches are oriented in the *y*-direction, the currents in the upper and lower parts of each branch are in reverse directions. Moreover, the current amplitudes on the branches are smaller than the amplitudes of **J***_x_*_1_, **J***_x_*_2_, and **J***_x_*_3_ on the central microstrip line along the *x* direction, so the polarization mode is still *x*-directional linear polarization. Because the electrical dimension of the narrow slot width is small, it can be assumed that those reverse currents **J**_0_ cancel out with each other, avoiding the generation of unwanted distribution modes.

### 2.4. Parametric Studies

To analyze the impact of several key structural parameters on antenna performance, parametric studies and optimization are carried out. In this work, the sequential single-parameter iterative optimization method is adopted, which is a conventional and widely accepted approach in antenna design. The −10 dB impedance bandwidth is set as the primary optimization objective, while port isolation, broadside gain, and overall size are taken as comprehensive constraints. Since radiation aperture parameters (S_1_, S_2_) and feed matching parameters have weak coupling, a two-stage optimization strategy is adopted: the radiation structure dimensions are determined first to define basic resonant characteristics, and then feed structure parameters are adjusted to optimize impedance matching, which effectively reduces the complexity of multi-parameter coupling. The final structural parameters are obtained through multiple rounds of iterative optimization to achieve optimal comprehensive performance.

**Figure 5:**
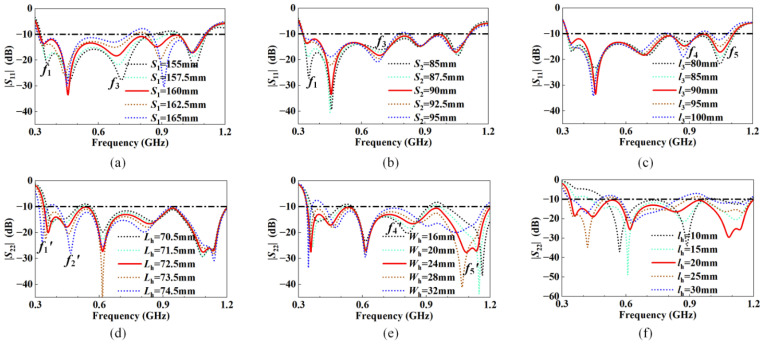
Parametric studies for the proposed antenna: (**a**) *S*_1_ and |*S*_11_|; (**b**) *S*_2_ and |*S*_11_|; (**c**) *l*_3_ and |*S*_11_|; (**d**) *L*_h_ and |*S*_22_|; (**e**) *W*_h_ and |*S*_22_|; (**f**) *l*_h_ and |*S*_22_|.

Figure 5a presents the simulated |*S*_11_| for different side lengths *S*_1_ of the wide slot. Among the investigated values, *S*_1_ = 160 mm yields the widest −10 dB impedance bandwidth of 0.32–1.11 GHz. Physically, enlarging *S*_1_ elongates the current path along the modified wide slot, thereby driving the low-frequency resonance *f*_1_ toward lower frequencies. A concurrent downward shift of the mid-frequency resonance *f*_3_ is also observed, which is primarily attributed to the enhanced electromagnetic coupling between the square ring and the wide slot.

Figure 5b illustrates the influence of the outer side length *S*_2_ of the square ring on |*S*_11_|. An increase in *S*_2_ moderately suppresses the low-frequency resonance *f*_1_, an effect mainly governed by the mutual coupling between the modified wide slot and the square ring. Meanwhile, the lengthened current path around the square ring pulls the mid-frequency resonance *f*_3_ downward. Balancing bandwidth extent and resonance depth at the low-frequency end, *S*_2_ = 90 mm is identified as optimal, offering the widest bandwidth of 790 MHz together with a well-defined low-frequency resonance.

The role of the monopole-like feeding structure is examined in Figure 5c through |*S*_11_| versus the branch length *l*_3_. Unlike *S*_1_ and *S*_2_, variations in *l*_3_ have a marginal effect on the low- and mid-frequency resonances *f*_1_, *f*_2_, and *f*_3_, while predominantly governing the high-frequency resonances *f*_4_ and *f*_5_. Increasing *l*_3_ shifts both high-frequency resonances downward; however, inter-branch coupling causes their depths to vary in opposite trends. To preserve deep high-frequency resonance, *l*_3_ = 90 mm is adopted.

The simulated |*S*_22_| for different lengths *L*_h_ of the rectangular slot etched on the modified wide slot is depicted in Figure 5d. As *L*_h_ increases, the current path of the modified wide slot becomes longer, and a significant shift of the low-frequency resonance *f*_1_′ toward lower frequencies can be observed. The optimal *L*_h_ is 72.5 mm, achieving the wide bandwidth and deep resonance depth.

Figure 5e depicts the simulated |*S*_22_| for different widths *W*_h_ of the rectangular slot. It can be observed that as *W*_h_ increases, the low-frequency resonance shifts toward lower frequencies with a decrease in depth, which is attributed to the fact that a larger rectangular slot width is unfavorable for the excitation of the meandered microstrip line. Meanwhile, an increase in *W*_h_ causes the high-frequency resonances *f*_4_′ and *f*_5_′ to converge toward the middle. Therefore, the optimal *W*_h_ is 24 mm.

The length *l*_h_ of the meandered microstrip line dominates the impedance matching of the antenna across its entire operating band, as illustrated by the |*S*_22_| simulations in Figure 5f. Variations in *l*_h_ modulate the coupling strength between the meandered microstrip line and the radiating structure, which subsequently influences the impedance of the antenna across the entire operating band. For optimal performance, *l*_h_ = 20 mm is selected, which yields the maximum bandwidth of 860 MHz together with deep resonance across all operating frequencies.

## 3. Antenna Performance and Discussion

**Figure 6:**
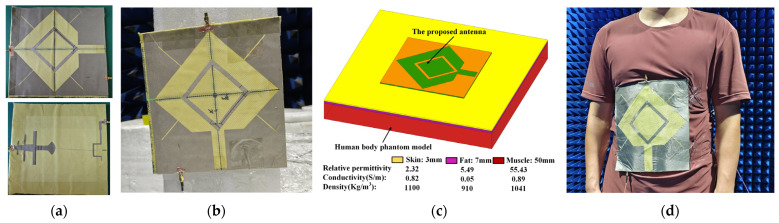
Experimental photographs and the human model: (**a**) antenna prototype; (**b**) measurement setup in free space; (**c**) three-layer human body cubic model; (**d**) measurement setup on human body.

The fabricated antenna prototype and measurement setup are shown in Figure 6. The gray patterns on the top and bottom layers were made of the flexible conductive fabric, and the yellow substrate was made of the Kevlar fabric. Benefiting from the all-textile flexible structure, the antenna is inherently suitable for integration with professional wearable scenarios including industrial workwear, emergency rescue jackets, and outdoor operation vests, and also has great potential for seamless integration with daily apparel. In the current research stage, the antenna is attached to clothing via adhesive tape, which is a standard verification scheme in the prototype phase of wearable antennas. This scheme can eliminate additional variables introduced by integration processes and accurately evaluate the core electromagnetic performance of the antenna itself. Benefiting from the all-textile, low-profile, and flexible structural features, the proposed antenna is inherently compatible with mainstream garment integration methods including sewing and thermal pressing. These integration processes only apply slight fixation on the edge of the antenna and do not significantly change the electromagnetic characteristics of the radiating structure. Systematic performance verification of garment integration prototypes belongs to engineering application research, which will be carried out in our follow-up work. The Kevlar fabric exhibits excellent hydrophobicity and temperature stability, whose performance is barely affected by ambient moisture and structural bending. While the potential irreversible effects of water washing on its electromagnetic and mechanical properties remain to be further investigated, its favorable temperature stability makes dry cleaning a feasible and applicable cleaning method for the clothing integrated with the proposed antenna.

It is worth noting that the physical size of the antenna is determined by its ultra-low operating frequency down to 300 MHz, and the design exhibits excellent miniaturization expansion potential in accordance with the electromagnetic scaling principle for commercial wearable application requirements at higher operating frequencies. In order to facilitate welding of the SMA connectors, several pieces of copper foil were pasted at specific positions. The entire pattern of the proposed antenna prototype was manufactured. To account for human body loading effects, a three-layer cubic human chest model (Figure 6c) was constructed. A 5-mm spacing was maintained between the antenna and the chest to simulate real-world wearing conditions.

### 3.1. S-Parameters

**Figure 7:**
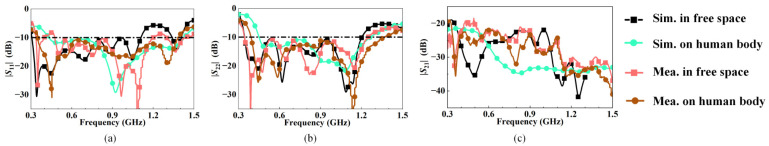
Simulated and measured reflection coefficients and port isolation of the proposed antenna: (**a**) |*S*_11_|; (**b**) |*S*_22_|; (**c**) |*S*_21_|.

Figure 7 shows the simulated and measured |*S*_11_|, |*S*_22_|, and |*S*_21_| of the proposed antenna in free space and on the human body. In free space, the measured −10 dB impedance bandwidths for the two ports are 1.1 GHz (0.34–1.44 GHz, 123.6% fractional bandwidth) and 0.89 GHz (0.37–1.26 GHz, 109.2%), respectively. Despite minor in-band fluctuations slightly above −10 dB, these measurements agree well with the simulated bandwidths of 0.79 GHz (0.32–1.11 GHz, 110.5%) and 0.86 GHz (0.34–1.20 GHz, 111.7%). On the human body, the measured bandwidths are 119.1% (0.35–1.38 GHz) for Port 1 and 115.9% (0.39–1.37 GHz) for Port 2. The discrepancies between simulated and measured results are caused by the combined effect of multiple factors, and the main error sources are qualitatively analyzed as follows. First, the surface resistance of the conductive fabric introduces additional conductor loss, which mainly affects radiation efficiency and has negligible influence on resonant frequency and impedance matching. Second, the batch tolerance of the dielectric constant and loss tangent of the Kevlar substrate is one of the main sources of the slight shift of resonant frequencies. Third, the SMA connectors and copper foil welding areas introduce parasitic parameters, which mainly affect the smoothness of impedance matching in the high-frequency band. Fourth, the simplified three-layer homogeneous human body model differs from the electromagnetic parameter distribution of real human tissues, which contributes to the deviation of on-body performance results. Overall, the deviation between simulation and measurement is within an acceptable range, which verifies the reliability and robustness of the proposed design. Figure 7c confirms that the measured isolation between the two ports exceeds 19 dB in the cases of both free-space and human-body conditions.

### 3.2. Radiation Patterns, Gains and Efficiency

**Figure 8:**
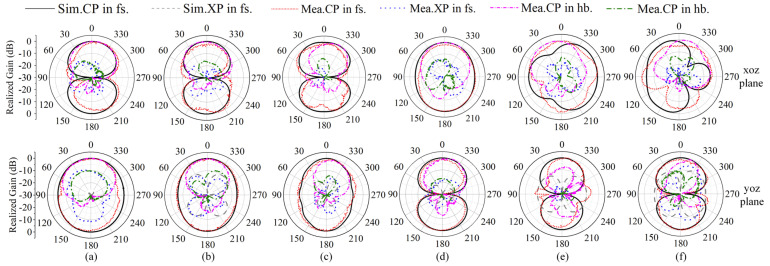
Normalized simulated and measured radiation patterns of the antenna: at (**a**) 0.35 GHz, (**b**) 0.65 GHz, and (**c**) 1.15 GHz for Port 1 excitation; at (**d**) 0.35 GHz, (**e**) 0.65 GHz, and (**f**) 1.15 GHz for Port 2 excitation. In the legend, fs. represents free space and hb. represents the human body.

Figure 8 shows the simulated and measured normalized radiation patterns of the proposed antenna in free space and on the human body. Measurements confirm that across the entire operating band, the antenna exhibits quasi-omnidirectional radiation patterns in free space and broadside patterns on the human body. In free space, the simulated average gains of the proposed antenna are 3.88 dBi for Port 1 and 3.74 dBi for Port 2, and the measured average gains within the operating band are 3.55 dBi for Port 1 and 2.76 dBi for Port 2. Moreover, the measured results indicate peak gains of 5.72 dBi (Port 1 at 0.92 GHz) and 4.63 dBi (Port 2 at 0.75 GHz). When placed on the human body, the average gains are 4.21 dBi (Port 1) and 3.54 dBi (Port 2), which can be attributed to the reflection-enhancing effects of body loading. The proposed antenna achieves an average FBR of more than 17 dB. The antenna achieves a radiation efficiency of 89.77% and 85.33% (in free space) for Port 1 and Port 2, respectively. There is good agreement between the simulated and measured results, and the differences between the results are mainly due to the fabrication and measurement errors.

It should be noted that, in the lower UHF band of 400–700 MHz, the electromagnetic wavelength is comparable to the curvature scale of the human torso. When the antenna is placed on the body surface, a portion of the radiated energy couples to human tissues and propagates tangentially along the body surface in the form of surface waves and creeping waves, which introduces additional coupling loss and affects the overall radiation efficiency. Meanwhile, the reflection effect of human tissues enhances the radiation intensity in the broadside direction, leading to an improved broadside gain compared with the free-space scenario. This combined effect is consistent with the simulation and measurement results presented in this section. The proposed dual-polarized ultrawideband design optimizes impedance matching and radiation aperture distribution, which effectively increases the proportion of energy radiated into free space and improves the transmission reliability of off-body communication links.

### 3.3. Bending Effects

**Figure 9:**
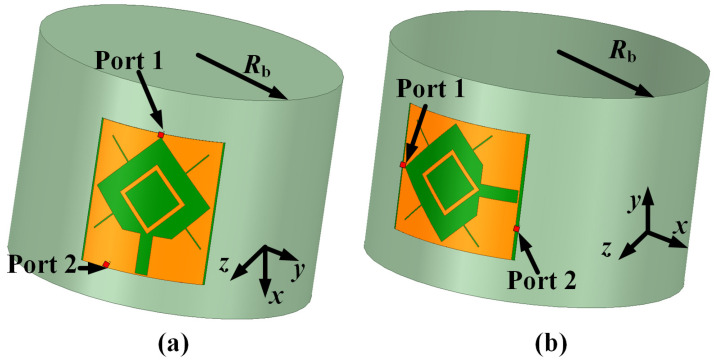
Illustration of the proposed antenna bent along: (**a**) *x*-axis; (**b**) *y*-axis with the bending radius *R*_b_.

**Figure 10:**
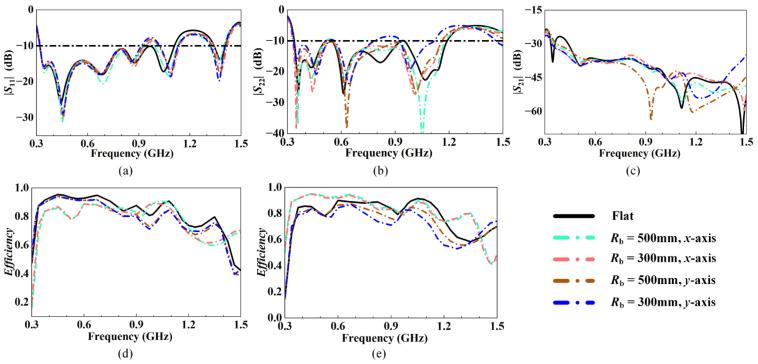
Simulated bending effect on *S*-parameter and efficiency: (**a**) |*S*_11_|; (**b**) |*S*_22_|; (**c**) |*S*_21_|; (**d**) efficiency for Port 1 excitation; (**e**) efficiency for Port 2 excitation.

**Figure 11:**
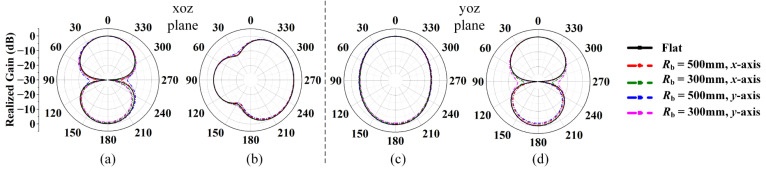
Simulated bending effect on the normalized radiation patterns: (**a**,**c**) Port 1 excited; (**b**,**d**) Port 2 excited.

As shown in Figure 9, we have analyzed the effects of bending along the *x*- or *y*-axis (with bending radius *R*_b_ of 300 mm and 500 mm) on the antenna performance. As shown in Figure 10a, when Port 1 is excited, bending along the *x*-axis or *y*-axis has essentially the same impact on impedance matching. Seen from Figure 10b, when Port 2 is excited, bending along the *y*-axis has a slightly greater impact on impedance matching, while bending along the *x*-axis improves impedance matching to a certain extent. The reason is mainly due to the difference in feeding mechanism for the Port 1 and Port 2 modes, respectively. Therefore, regardless of bending along the *x*-axis or *y*-axis, the degree of deterioration to the Port 1 mode is relatively slight and basically consistent. When bending along the *x*-axis, the impedance matching for the Port 2 mode is improved to some extent; when bending along the *y*-axis, however, the impedance matching for the Port 2 mode is slightly deteriorated. It can be observed that bending causes a slight decrease in the resonant frequency, and the greater the bending degree, the more obvious the frequency shifts. Seen from Figure 10c, bending improves the isolation between those two ports. This bending insensitivity stems fundamentally from the antenna’s ultrawideband (UWB) operation, whose inherent low Q-factor reduces the resonant structure’s sensitivity to bending-induced geometric perturbations. From Figure 10d, when Port 1 is excited, the efficiency decreases slightly. From Figure 10e, when Port 2 is excited, bending along the *x*-axis slightly increases the efficiency, whereas bending along the *y*-axis slightly decreases it. Figure 11 demonstrates the effect of bending on the normalized radiation patterns. Taking the mid-band frequency of 0.7 GHz as an example, it can be seen that bending has a minimal effect on the radiation patterns. Table 1 shows the simulated average gains under different bending cases, which also indicates minor influence of bending. The results indicate that under these bending conditions, exciting either the Port 1 or Port 2 mode yields stable performance.

### 3.4. SAR Results

**Figure 12:**
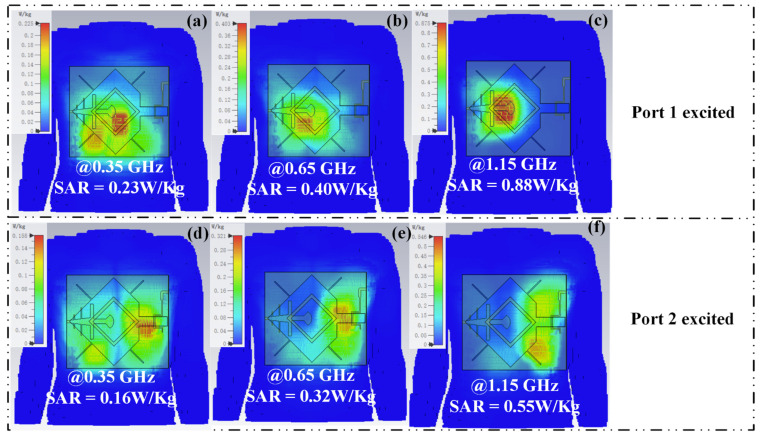
SAR analysis of the proposed antenna on the human body at (**a**) 0.35 GHz, (**b**) 0.65 GHz, and (**c**) 1.15 GHz for Port 1 excitation; at (**d**) 0.35 GHz, (**e**) 0.65 GHz, and (**f**) 1.15 GHz for Port 2 excitation (the gap between the antenna and the body is 5 mm).

The SAR is used to evaluate the electromagnetic safety of the antenna. The SAR with a low value is required for human health when a wearable antenna is used. SAR calculations follow the formulas in [40,48]. For an input power of 0.5 W, the antenna’s SAR levels are calculated by exciting ports 1 and 2, respectively. Per the IEEE C95.1 standard, the maximum 10g-averaged SAR in human tissue must be less than 2 W/kg. As shown in Figure 12, the SAR levels are significantly below this safety limit.

### 3.5. Wireless Transmission Quality Assessment

**Figure 13:**
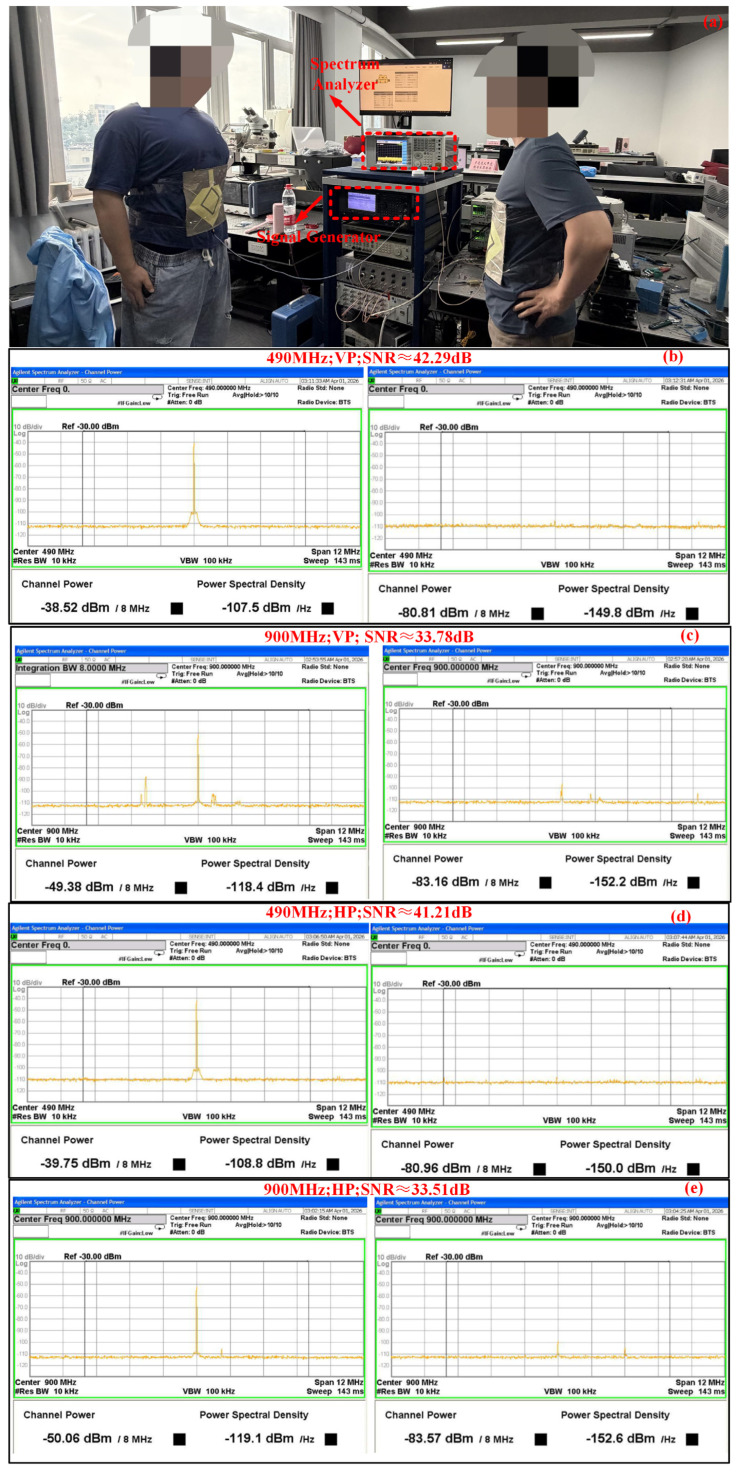
Wireless transmission experiment: (**a**) measurement setup; measured SNR results under (**b**) 490 MHz with VP, (**c**) 900 MHz with VP, (**d**) 490 MHz with HP, and (**e**) 900 MHz with HP, where the left and right panels in (**b**–**e**) show the spectra with and without the transmitted signal, respectively.

To validate the proposed antenna’s capability for off-body communications in IoT-based WBAN scenarios, a practical wireless transmission experiment was implemented. The measurement setup is shown in Figure 13. Two proposed wearable textile antennas were employed as the transmitting and receiving terminals, respectively. The transmitting antenna was connected to a signal generator (PSG Analog Signal Generator E8527D from KEYSIGHT), while the receiving antenna was conformally placed on the user’s chest and connected to a spectrum analyzer (MXA Signal Analyzer N9020A from KEYSIGHT). The two antennas are positioned approximately 2 m apart to emulate a typical off-body communication link. The signal generator was configured to transmit a continuous wave signal at a power level of −10 dBm, and the integration bandwidth of the spectrum analyzer was set to 8 MHz. Two representative IoT frequency bands, 490 MHz and 900 MHz, are selected for the evaluation. At each frequency, the SNR was measured under both horizontal polarization (Port 1 excited) and vertical polarization (Port 2 excited) states of the proposed antenna. When the signal generator was turned off, the noise power spectral density was captured by the spectrum analyzer. When the signal generator was activated, the combined signal-plus-noise power spectral density was recorded. The detailed SNR results under different frequency bands and polarization configurations are presented in Figure 13. The results demonstrate that the proposed antenna achieves high SNR values across both tested frequencies and polarization modes, confirming its capability to effectively receive the desired signal with a substantial power margin above the noise floor, and highlighting its significant potential for reliable off-body communications.

Table 2 compares the radiation performance of the proposed antenna with that of typical state-of-the-art wearable antennas. Compared to the multiband wearable antennas reported in [28,29,32] and ultrawideband wearable antennas reported in [37,38,41], the proposed antenna features the key merit of ultrawideband operation, dual-polarization, and more compact size. Compared to the multiple-polarized wearable antennas reported in [42,43,44], the proposed antenna offers significantly wider operating bandwidths for both polarization modes, while featuring a smaller area and lower profile. Most of the above-mentioned wearable antennas are designed to operate at the 2.45 GHz and 5.8 GHz bands. Compared with the triband dual-polarized antenna designed for the Cospas-Sarsat (C-S) search-and-rescue satellite system operating at 406 MHz reported in [7], the proposed antenna achieves wider operating bandwidth, higher gain, and more compact dimensions. Hence, the proposed antenna exhibits notable comprehensive performance advantages in terms of bandwidth, size, and polarization diversity in the low UHF band. In summary, the proposed antenna is very well-suited for wearable off-body communication applications.

### 3.6. Limitations of This Work

Despite the favorable electromagnetic performance achieved by the proposed antenna, there are still objective limitations in the current prototype-stage research, which are summarized as follows:

On-body test scope: A simplified three-layer homogeneous human chest model and static on-body tests are adopted in this work. Dynamic on-body scenarios with different body shapes, wearing positions and motion states are not covered, and large-scale multi-subject tests will be carried out in follow-up research.

Mechanical reliability: Only the electromagnetic performance under fixed bending radii is verified. Long-term mechanical durability evaluations such as repeated bending fatigue and cyclic tensile deformation have not been carried out systematically.

Environmental robustness: Environmental aging tests including washing cycles and sweat erosion have not been conducted. The long-term performance stability of the antenna in actual daily wearing scenarios needs further engineering verification.

The above limitations do not affect the core contribution of this work in antenna structural design and principle verification. Relevant systematic engineering validations will be gradually improved in our follow-up research.

## 4. Conclusions

In this article, we propose a compact ultrawideband dual-polarized wearable textile antenna for off-body communications in IoT-based WBAN scenarios. The antenna utilizes a square-ring loaded wide slot as the basic radiation structure. Oppositely placed advanced monopole-like microstrip line with parallel branches and meandered microstrip line are used as the feeding networks for bandwidth extension, dual-linear polarization, and improved port isolation. Four narrow slots are orthogonally etched around the wide slot to extend the current path and miniaturize the antenna sizes. Also, the performance of the proposed antenna on the human body is validated. Finally, the antenna prototype was manufactured manually and measured. The measured impedance bandwidths are 119.1% and 115.9% for the Port 1 and Port 2 modes on the human body (123.6% and 109.2% in free space), respectively. The port isolation is more than 19 dB. A compact size of 0.30 × 0.28 × 0.0035 *λ*_l_^3^ is realized. The quasi-omnidirectional beam patterns of the proposed antenna are transformed into unidirectional beams because of the human body loading effects. The antenna achieves a high FBR exceeding 17 dB without an additional reflector. For a typical input power of 0.5 W, the SAR remains within the safety limit. Wireless transmission experiments at representative IoT bands further demonstrate that the proposed antenna supports reliable off-body links with excellent signal-to-noise ratios for both polarizations.

## Figures and Tables

**Table 1:** Simulated average gain under different bending cases.

Cases		Flat	*R*_b_ = 500 mm *y*-Axis	*R*_b_ = 300 mm *y*-Axis	*R*_b_ = 500 mm *x*-Axis	*R*_b_ = 300 mm *x*-Axis
Average Gain (dBi)	Port 1 excited	3.88	4.01	4.11	3.86	3.92
	Port 2 excited	3.74	3.59	3.43	3.77	3.98

**Table 2:** Performance comparison of the proposed antenna with other corresponding wearable antennas.

Refs	Antenna Type	Impedance Bandwidth @f_c_ (GHz)	Polarization	Peak Gain * (dBi)	SAR (W/Kg)	Size (*λ*_l_^3^)
[28]	Monopole PIFA	12% @2.45 44% @5.80	Single-LP	1.20 5.70	0.211 0.066	0.47 × 0.49 × 0.0699
[29]	Loop Patch AMC	9% @2.45 10% @5.80	Single-LP	6.00 8.20	0.37 0.22	0.83 × 0.83 × 0.0397
[32]	Monopole Annular-Half-Disk	6.9% @2.45 5.9% @5.80	Single-LP	3.90 3.50	0.86 0.58	0.51 × 0.51 × 0.0300
[37]	Monopole AMC	34% @5.80	Single-LP	6.12	1.54	1.46 × 0.97 × 0.0516
[38]	SIW Slot	26% @8.00	Single-LP	5.20	0.305	1.37 × 0.99 × 0.0775
[41]	Quasi-Yagi ME Dipole	68% @2.65	Single-LP	8.23	N.A.	0.50 × 0.33 × 0.0046
[42]	Monopole Crossed Dipole	7% @2.45 52% @5.80	LP CP	2.20 8.60	1.04 0.29	0.49 × 0.49 × 0.0773
[43]	Patch Series-Fed	33.2%@5.54	RHCP LHCP	1.20 3.20	N.A.	1.08 × 1.08 × 0.0078
[44]	CRLH-TL	13.1%/10.8% /35.3%/5.3% (|*S*_11_| < −6 dB)	Quad-LP	−0.9/1.3 /2.6/1.6	1.75/0.51 /0.30/0.53	*π* × 0.13^2^ × 0.0500
[7]	Annular Patch	3.4% @0.406 3.3% @1.227 4.6% @1.575	LP CP CP	−1.97 4.27 3.37	0.032 0.023 0.037	0.35 × 0.35 × 0.0110
This work	Wide Slot Square-Ring Resonant Feed	123.6%@0.865 109.2%@0.88	V-LP H-LP	5.72 4.63	0.88 0.55	0.30 × 0.28 × 0.0035

* To be fair, the peak gains of the antennas in free space are compared. “N.A.” means “these data are not available in the work”. “f_c_” means “center frequency”.

## Data Availability

All data generated or analyzed during this study are included in this article.
